# Evaluation of In Vitro Antimicrobial, Cytotoxic, Thrombolytic, and Antiarthritic Property of Different Parts of Bari Orchid

**DOI:** 10.1155/2024/8148610

**Published:** 2024-04-30

**Authors:** Md. Samsur Rahaman, Md. Saifur Rahaman, Shah Md. Marzuk Hasnine, Salma Sultana, Md. Abdul Quaiyum Bhuiyan, Mohammad Shahriar Kabir, Md. Abdul Bari, Jahid M. M. Islam, Md. Ismail Hossain, Mubarak A. Khan

**Affiliations:** ^1^Sonali Bag Project, Bangladesh Jute Mills Corporation, Ministry of Textiles and Jute, Dhaka, Bangladesh; ^2^Research Lab, Echotex Ltd., Gazipur, Bangladesh; ^3^Institute of Nuclear Science and Technology, Bangladesh Atomic Energy Commission, Dhaka, Bangladesh; ^4^Department of Chemistry, Primeasia University, Dhaka 1213, Bangladesh; ^5^Institute of Food and Radiation Biology, Bangladesh Atomic Energy Commission, Dhaka, Bangladesh; ^6^School of Science, Monash University, Sunway Campus, Subang Jaya, Selangar 47500, Malaysia; ^7^Agriculture Statistics Division, Bangladesh Rice Research Institute, Gazipur 1701, Bangladesh

## Abstract

Many different herbal extracts have historically been utilized to treat microbe-induced infections, injuries, cancer, thrombosis, and arthritis. The purpose of this study was to determine the antibacterial, cytotoxic, in vitro thrombolytic, and in vitro antiarthritic properties of ethanolic extracts of stem and seed of Bari orchid 1 (BO) plant. This orchid plant was developed by the Bangladesh Agriculture Research Institute (BARI) in Gazipur. Fourteen microbes were employed in the antimicrobial investigation, and samples of orchids were compared to ciprofloxacin as a reference. The BO/seed extract was found to possess more antibacterial activity. The lethality test of brine shrimps was used to assess the LC_50_ values. The BO/stem extract exhibited a higher cytotoxicity potential, in comparison to the BO/seed extract. Two concentrations (1000 and 100 ppm) and two incubation times (24 hours and 1.5 hours) were used to assess the thrombolytic activity of the extracts. Regarding the thrombolytic effect, the BO/stem extract has demonstrated greater promise. Furthermore, the herbal extract's antiarthritic activity was investigated at four different concentrations, and the results were evaluated in comparison with those of diclofenac sodium. When comparing BO/stem extract to other extracts, the greatest values for protein denaturation were obtained.

## 1. Introduction

Since the beginning of time, herbal medicines have been used to treat a wide range of illnesses [1]. From ancient times, these medicines were formulated by the therapeutic expertise of generations of physicians who had been practicing for centuries [2]. Nature has given us a gift in the form of medicinal plants that can be used to treat and prevent diseases [3]. Orchids are attractive flowers that have high value in society as ornamental plants. In addition, orchids are also known for their usage, especially in traditional medicine [4].

**Figure 1:**
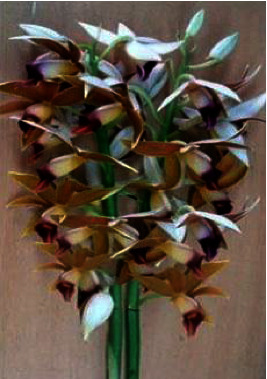
Bari orchid 1.

Bari orchid 1 (BO) is an orchid that possesses a long flower stalk with 12 to 15 florets, the petal's outer side is creamy white, and the petal's inner color is reddish-brown with a distinct maroon color tip and a golden yellow lip (Figure 1). The flower has a sweet fragrance. This orchid is developed in Bangladesh Agriculture Research Institute (BARI)situated in Jaintapur, in the Sylhet district of Bangladesh [5]. So far, no conventional applications have been established. Thus, traditional applications have no effect on the phytochemical and pharmacological properties.

Antimicrobial resistance is increasing globally (particularly in developing countries) as a result of the overuse and misuse of antibiotics [6]. As a result, antibiotic resistance causes 700, 000 to several million deaths per year [7, 8]. The World Health Organization says that the threat of antimicrobial resistance needs to be fixed as soon as possible [9]. Natural antimicrobial compounds that can treat microbial infections well have been found in a lot of medicinal plants [10]. Medicinal plants would be the best source to acquire various drugs, as declared by the World Health Organization [11]. However, there are some limitations to old and newly introduced antimicrobial agents, e.g., short life expectancy, higher side effects, etc. [12, 13]. Since resistant clinical isolates are quickly spreading around the world, it is most important to find new antimicrobial agents.

Thrombosis (blood clot) is a critical medical condition that is usually found in the veins of the arms, pelvis, thigh, lower leg, etc. To retrieve from this leads to disability, illness, and even death [14]. Cardiovascular disease is the leading global cause of death, accounting for more than 17.9 million deaths in 2015, with a projected annual death rate of more than 23.6 million by 2030 [15]. The most common examples of thrombolytic agents are tissue plasminogen activator, streptokinase, urokinase, antistreptokinase, etc. These main agents dissolve the clots [16]. Thrombolytic agents do not work without adverse effects, such as bleeding, and to work best, a large quantity of fibrin-specific medicines are required [17]. Significant efforts have been made to discover and develop thrombolytic, antithrombotic, antiplatelet, and anticoagulant agents from natural constituents, e.g., various animal and plant sources, which will open new horizons to encourage the advancement of proper alternative thrombolytic therapy [18].

One of the autoimmune diseases, arthritis, is recognized by the co-occurrence of pain, stiffness, and swelling. It is brought on by synovial joint inflammation as a result of an immune-mediated reaction. Around the world, arthritis affects one in five elderly people [19]. NSAIDs, sulfasalazine, D-penicillamine, cyclophosphamide, cyclosporine, methotrexate, azathioprine, glucocorticoids, anakinra, etanercept, abatacept, and infliximab, among others, have been used to treat arthritis [20–22]. But there are some risks, such as gastrointestinal ulcers, stomatitis, problems with the heart and blood vessels, pulmonary toxicity, myelosuppression, hematologic nephrotoxicity, hepatic fibrosis, cirrhosis, diarrhea, and local reactions at the injection site [23]. Because of this, it is very important to make new antiarthritis medicines from medicinal plants that are cheap and have few side effects.

Secondary metabolites such as alkaloids, saponins, terpenoids, tannins, flavonoids, glycosides, inulin, steroids, terpenoids, phlorotannin, phenols, essential oils, resins, and naphthoquinone give plants their strong pharmacological properties. As treatments for diseases, these substances work by changing the way the body works [24, 25]. Because people don't know enough about safe dosages and because some plants have toxic byproducts, using local plants as a source of medicine may have some bad effects [26].

In the current study, the in vitro antimicrobial, thrombolytic, antiarthritic, and cytotoxicity effect of these orchid plant extracts were evaluated because there is no scientific evidence of the antimicrobial, thrombolytic, antiarthritic, and cytotoxicity potential of BO extracts.

## 2. Materials and Methods

### 2.1. Preparation of Extract

BO was collected from the Horticulture Division, Bangladesh Agriculture Research Institute, Gazipur, Bangladesh. The plants were washed with ethanol and dried separately at room temperature (22 ± 0.5°C) for 15 days and grounded to powder form. Then, 250 g of ground plant parts were soaked in 1.0 L ethanol for seven days using screw-capped reagent bottles. Filtrates were obtained through the Buchner funnel by Whatman filter paper No. 11 and concentrated by rotary evaporator (operating below 40°C and lower pressure). Different plant extracts were then suspended in distilled water and mixed vigorously by a vortex mixer to make the desired concentration.

### 2.2. Phytochemical Screening

Phytochemical investigation of BO was done through the standard procedures as mentioned in Evans; Harbone and Rahaman et al.. Notably, macronutrients and secondary metabolites were tested for in the extracts [27–29].

#### 2.2.1. Test for Alkaloids

Mayer's Test: 0.2 g of each extract was taken in test tubes and boiled on a steam bath after adding 5 mL of 2% HCl. Then, the sections were filtered. After that, 1 mL of filtrates was taken in separate test tubes, and two drops of Mayer's reagent were added. Precipitation with a creamy white color indicates the presence of alkaloids.

#### 2.2.2. Test for Flavonoids

0.2 g of each extract and 10 mL ethyl acetate were taken in test tubes. Then, the test tubes were heated at 100°C for 3 minutes using a water bath. After that, each extract was filtered, and filtrates were drawn for the below tests:

1. Ammonium test: 4 mL of the filtrates were shaken vigorously with 1 mL of 1% diluted ammonia. Then, the layers were allowed to separate. The yellow coloration of an ammonia layer proves the presence of flavonoids.–colored
2. Aluminum chloride test: 4 mL of each filtrate was shaken vigorously with 1 mL of 1% aluminum chloride and allowed to sit for precipitation. The formation of yellow-colored precipitation justifies the presence of flavonoids.

#### 2.2.3. Test for Glycosides

Keller–Kiliani Test: 0.5 g of each extract was dissolved in 2 mL of chloroform. Then, the mixture was filtered, and each filtrate was assigned to separate test tubes. After that, those filtrates were evaporated to dryness by the application of heat. After drying all filtrates, 1 mL of glacial acetic acid and three drops of 5% w/v ferric chloride were added to each test tube. Afterwards, 1 mL of fuming sulfuric acid was cautiously added to the side of the test tubes. The appearance of a bluish-green color in the higher layer confirms the presence of glycosides.

#### 2.2.4. Test for Steroids

0.2 g of each extract was taken in test tubes and dissolved in 2 mL of chloroform. Then the following tests were done.

1. Salkowski test: Each test tube received 2 mL of fuming sulfuric acid and was shaken for a few minutes. The development of red coloration in the chloroform layer indicates the presence of steroids.
2. Liebermann–Burchard test: Ten drops of acetic anhydride were added to the test tubes and mixed properly. Then, 2 mL of concentrated sulfuric acid was added to the test tube. The presence of steroids is confirmed by the transient greenish coloration.

#### 2.2.5. Test for Terpenoids

0.2 g of the extracts were taken in test tubes, dissolved in 2 mL of chloroform, and evaporated to dryness. Then, 2 mL of concentrated sulfuric acid was added to the test tubes and heated for about 2 minutes. The development of grayish color indicates the presence of terpenoids.

#### 2.2.6. Test for Saponins

Froth Test: 0.1 g of each extract was taken in test tubes and diluted with 15 mL of distilled water, and the mixture was shaken vigorously for 15 minutes. The formation of a 1 cm layer suggests the presence of saponins.

#### 2.2.7. Test for Phenols

FeCl_3_ Test: 0.2 g of each extract was taken in test tubes and boiled with 5 mL of 45% ethanol for 5 min. Then, the mixture was cooled and filtered. After that, 5 mL of distilled water and three drops of 5% FeCl_3_ (w/v) were added with 1 mL of the filtrate. A transient greenish to black coloration confirms the presence of phenols.

#### 2.2.8. Test of Tannins

Gelatin Test: 0.2 g of extracts and 3 mL of 1% gelatin containing 10% NaCl (a few drops) were added to test tubes. The presence of white precipitate suggests the presence of tannins.

#### 2.2.9. Test for Carbohydrates

Molisch's Test: 0.1 g of extracts and 5 mL of distilled water were taken in each test tube. Then, those were shaken vigorously and filtered. To the filtrates, five drops of Molisch reagent were added and shaken vigorously again. After that, 1 mL of fuming sulfuric acid was carefully added to the test tubes. The appearance of a brown ring at the interface indicates the presence of carbohydrates.

#### 2.2.10. Test for Proteins

1. Biuret's Test: 3 mL of extracts, 1 mL of 4% w/v sodium hydroxide, and 1 mL of 1% w/v copper sulfate were taken in test tubes. The change in color of the extracts from blue to violet or pink proves the presence of proteins.
2. Xanthoproteic Test: 3 mL of each extract and 1 mL of concentrated sulfuric acid were taken in test tubes. Firstly, white precipitate forms, which then turns yellow on boiling; orange precipitation forms after the addition of 1 mL ammonium hydroxide, confirming the presence of proteins, e.g., tyrosine and tryptophan.

### 2.3. Antimicrobial Screening

The disc diffusion method (CLSI guideline) was used for screening the antibacterial and antifungal properties of plant extracts as described by Daoud et al. [30, 31]. Five Gram-positive bacterial strains (*Staphylococcus aureus* ATCC-25923, *Sarcina lutea* ATCC-9341, *Bacillus subtilis* ATCC-6633, *Bacillus cereus* ATCC-11778, and *Bacillus megaterium* ATCC-14581), Gram-negative bacteria (*Escherichia coli* ATCC-25922, *Pseudomonas aeruginosa* ATCC-49189, *Vibrio parahaemolyticus* ATCC-17802, *Salmonella typhi* ATCC-14028, *Shigella boydii* ATCC-9207, *Vibrio mimicus* ATCC-33653, *Salmonella paratyphi* ATCC-9150, and *Shigella dysenteriae* ATCC-13313), and fungi (*Aspergillus niger* ATCC-16404, *Candida albicans* ATCC-10231, and *Saccharomyces cerevisiae* ATCC-9763) were collected from the Pharmacy Department, University of Dhaka, Bangladesh. Those stains were used as pure culture and maintained on the nutrient agar medium (Oxoid, UK). The dried sterile Matricel filter paper discs (6.0 mm diameter, BBL, USA) were soaked in each extract (400 *µ*g/disc) dissolved in methanol. Then, those were dried to evaporate the residual methanol. Ciprofloxacin (5 *µ*g/disc) was used as the positive control, and the blank disc was used as the negative control. The sample discs, the standard antibiotic discs, and dried blank were placed on petri dishes containing nutrient agar medium consistently seeded in the test bacteria and fungi. Then those were kept in a refrigerator at 4°C for about 24 h. The upside of petri dishes was placed on the bottom to allow for adequate diffusion of materials from the discs to the surrounding agar medium. Then, each petri dish was inverted and placed in an incubator at 37°C for 24 hours. Their activity was measured by the diameter of the zone of inhibition, which was given in millimeters [32, 33]. This showed how well they stopped microorganisms from growing.

### 2.4. Brine Shrimp Lethality Bioassay

The brine shrimp lethality bioassay was used to predict that the ethanolic extract of BO would be cytotoxic [34, 35]. For the experiment, 1 mg of the extracts were dissolved in dimethylsulfoxide (DMSO) and solutions of varying concentrations (100, 50, 25, 12.5, 6.25, 3.13, 1.56, 0.78, 0.39, 0.19, and 0.09 mg/mL) were obtained by the serial dilution technique using simulated seawater. The solutions were then poured in the pre-labeled test tubes, which contained ten live brine shrimp nauplii in 5 mL simulated seawater. The test tubes were examined with a 3× magnifying glass, and the number of brine shrimp nauplii that survived after 24 hours was counted. The absence of controlled forward motion during 30 seconds of observation was defined as mortality [36]. The percentage of lethality was calculated for each extract and control from this data. Vincristine sulfate and DMSO were used as the positive and negative controls, respectively. The values of LC_50_ were found by plotting the logarithm of concentration against the percentage of deaths in Microsoft Excel Plus 2016 [37]. After 24 hours, the number of brine shrimp nauplii that were still alive was counted, and the following equation was used to figure out the percentage of deaths [34, 38]:

(1) $$\begin{array}{c} \text{Percent of mortality}=\left(\frac{\text{no}.\text{ of dead nauplii after }24\;h}{\text{initial no}.\text{ of live nauplii}}\right)\times 100. \end{array}$$

### 2.5. In Vitro Thrombolytic Activity

Thrombolytic properties were investigated according to the method disclosed in Prasad et al. [39, 40].

### 2.6. Standard Solution Preparation

To prepare a standard stock solution, commercially available lyophilized streptokinase (SK) in a vial containing 1500000 IU was collected and thoroughly mixed with 5 mL of sterilized distilled water. All chemical substances in this investigation were of analytical reagent grade.

### 2.7. Blood Collection

Sixty milliliters of blood was collected from 20 healthy human volunteers without a record of smoking, taking an oral contraceptive, or anticoagulant therapy.

#### 2.7.1. Procedure

The collected blood was allowed to clot in falcon tubes for around fifteen minutes. Then, the serum was separated from the blood using a centrifuge machine (Model: Remi-R-83 A) operated at 2000 rpm for 1 minute. After that, the serum was entirely separated without disturbing the clot, and reweighing each falcon tube was done to measure clot weight. All the falcon tubes containing clots were labeled correctly, and 100 *µ*l of different plant extracts, 100 *µ*l of SK (Positive Control), and 100 *µ*l of distilled water (Negative Control) were added to the falcon tubes. Then, falcon tubes were kept in an incubator at 37°C for 90 minutes and 24 hours to monitor the clot lysis. The dissolved clot was carefully taken out, and after 90 minutes and 24 hours of incubation, the gravimetric method was used to figure out the clot lysis percentage. This test was done in triplicate.

(2) $$\begin{array}{c} \%\text{ of clot lysis}=\left(\frac{\text{Weight of Released Clot}}{\text{Weight of Clot}}\right)\times 100. \end{array}$$

### 2.8. In Vitro Antiarthritic Activity (Egg Albumin Denaturation Method)

The method described in Pavithra et al. [41] was used to keep track of arthritic activities.

### 2.9. Phosphate Buffer Saline pH 6.4

In a 1000 mL volumetric flask, 8 g of sodium chloride, 0.2 g of potassium chloride, 1.44 g of disodium hydrogen phosphate, 0.24 g of potassium dihydrogen phosphate, and 800 mL distilled water were taken and mixed correctly. Then, the pH of the solution was adjusted to 6.4 using 1 N HCl and filled with the needed amount of distilled water, making up the mark of 1000 mL.

### 2.10. Procedure

To evaluate the antiarthritic activity, 2.8 ml of phosphate-buffered saline (pH 6.4), 2 ml of various plant extract concentrations (66.5, 125, 250, and 500 ppm), and 0.2 ml of fresh hen's egg albumin were taken in each test tube. 2 mL of distilled water was served as control. 2 mL of varying concentrations of diclofenac sodium (66.5, 125, 250, 500 ppm) were served as the standard. After that, the mixtures were incubated at 37 ± 2°C in a BOD incubator for 15 minutes. Then, the mixtures were heated at 70°C for 5 minutes in a water bath. After cooling the solutions, absorbance was measured at 660 nm by a UV-Vis spectrophotometer. As a control, phosphate-buffered saline was used. The percent inhibition of denaturation of proteins was calculated using the following formula:

(3) $$\begin{array}{c} \%\text{ inhibition of protein denaturation}=100\times \left[\frac{\text{Vt}}{V_{{}^{\circ}}-1},\right] \end{array}$$

where Vt = absorbance of test sample and *V*_°_ = absorbance of control.

The tests were done in triplicate.

### 2.11. Statistical Analysis

The data are presented as the standard deviation of three tests. With the aid of ANOVA, analysis was carried out through Dunnett's test. The values of *P* < 0.001 and *P* < 0.05 were considered significant.

## 3. Results and Discussion

### 3.1. Preliminary Phytochemical Screening

For the qualitative determination of the phytochemicals of ethanolic extract of the different parts of *Bari orchid 1*, these extracts were subjected to preliminary phytochemical screening according to standard procedures. The phytochemicals discovered are listed in Table 1. The positive signs of the phytochemicals confirm the presence of saponins, steroids, glycosides, terpenoids, carbohydrates, phenols, flavonoids, tannin, alkaloids, and proteins in the plant extracts (Table 1).

### 3.2. UV-Visible Spectrophotometric Analysis

The identification of phytoconstituents found in the plant extracts was accomplished using UV-Vis (Shimadzu, Model UV-2401 PC) analysis. The compounds with *σ*-bonds, *π*-bonds and lone pairs of electrons, chromophores, and aromatic rings were identified using the UV-visible spectra.  Table 2 shows the observed absorption bands for each plant extract.

**Figure 2:**
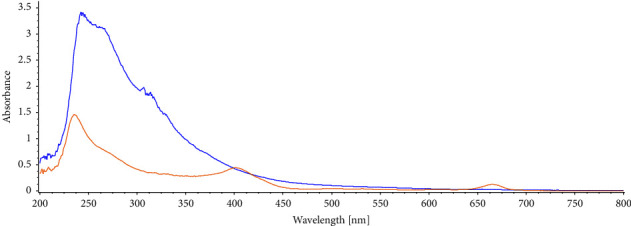
UV-visible spectra of different plants extract BO/seed/ethanol (blue) and BO/stem/ethanol (yellow).

In the UV-Vis spectra (Figure 2), the appearance of one or more peaks in the region from 200 to 400 nm is a clear indication of the presence of unsaturated groups and heteroatoms such as S, N, and O [42]. The UV-visible spectra of BO/seed/ethanol extract showed a peak at 242.1 nm that indicated the existence of flavonoids [43]. Peaks at 235.4 nm, 327.5 nm, 401.5 nm, and 665.4 nm in BO/stem/ethanol extract indicated the presence of steroid, terpenoid, and extended conjugation due to the presence of flavonoid.

### 3.3. ATR-FTIR Analysis

The functional group of the active components was found by looking at the peak value in the infrared part of the FTIR spectrum (Perkin Elmer, Spectrum-2). In Table 3, FTIR peaks for two different parts of BO extracts are reported.

**Figure 3:**
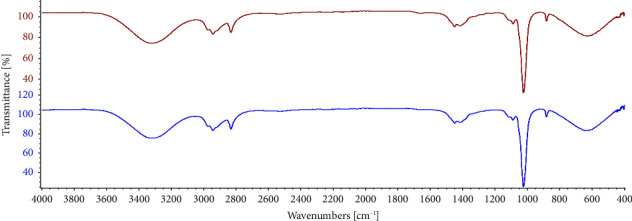
FTIR spectrum of different plant extracts BO/seed/ethanol (red) and BO/stem/ethanol (blue).

Figure 3 shows that the BO plant extracts of two different parts such as stem and seed revealed peaks at 881 cm^−1^ that correspond to the -CH_2_-rocking group which showed the existence of carbohydrate. Both parts of this plant extract showed peaks at 1023 cm^−1^ which correspond to the -C-O- group that revealed the presence of flavonoids and cardiac glycosides. BO/seed/ethanol plant extract presented peaks at 1449 cm^−1^ and 1654 cm^−1^ which represented the -CH_3_ group and -C=C- group, peak at 2943 cm^−1^ was responsible for -C-H- stretching of alkane that indicated the presence of terpenoids. In BO/stem/ethanol extract, there was no peak at 1654 nm which indicated that there is no presence of terpenoids which was further proved in the preliminary phytochemical screening section. Peaks at 2832 cm^−1^ were found in both parts of this plant extract, indicating -N-H- stretching that directed the presence of alkaloids. BO plant extracts of two different parts also displayed peaks at 3319 cm^−1^, 3325 cm^−1^ that correspond to the -OH- group which indicated the presence of phenolics and flavonoids [44].

### 3.4. Antimicrobial Activity

Antimicrobial drugs are substances, either naturally occurring or synthesized, that prevent or eradicate the growth of microorganisms such as bacteria, fungi, helminths, protozoa, and viruses. Antimicrobial drugs can be categorized based on how they combat microorganisms. These include compounds that depolarize cell membranes, prevent bacteria from forming cell walls, halt protein synthesis, inhibit nucleic acid synthesis, and disrupt metabolic processes. Some basic phytoconstituents are responsible for antimicrobial activity, e.g., alkaloids, flavonoids, and glycosides [45–48].

We tested the ethanolic extracts of two parts of BO for their antimicrobial activity against Gram-positive bacteria, Gram-negative bacteria, and fungi. Disc diffusion method was used for antimicrobial activity testing. All the plant's extract exhibited good antimicrobial properties against the microorganisms (Table 4).

Extracts demonstrated varying levels of activity against all microorganisms. The diameter zone of inhibition values for the extracts against all microorganisms were ≥10 mm. The zone of inhibition values ≥8 mm for medicinal plants is considered active. The diameter zone of inhibition varies from 10 mm to 14 mm. Based on the diameter of zones of inhibition, antimicrobial activity is categorized as inactive (<8 mm), less active (≥8 to <12 mm), moderately active (≥12 to <20 mm), and highly active (≥20 mm) [49]. The inhibition observed on the tested strains of bacteria shows that orchid extracts have a broad spectrum of activity of plant extracts against different strains of bacteria, which has been reported in other studies [50, 51].

For conventional and traditional medicine to be able to use the active compounds in the orchids that this study focused on, they need to be better understood and tested. Also, all of the tested extracts showed different levels of antimicrobial properties that fight infections caused by microorganisms.

### 3.5. Brine Shrimp Lethality Bioassay

Cytotoxic pharmaceuticals (also known as antineoplastics) are cancer-treating medications that introduce toxic substances to cells, blocking their reproduction or development. They can also treat rheumatoid arthritis and multiple sclerosis, among other conditions. Conventional cancer treatments are thought to work by killing only tumor cells or stopping their growth in a way that can't be undone. Cytotoxic medicines stop DNA from being made or damage DNA chemically, which kills tumor cells. The nauplii of brine shrimp are simple animals that can be used to measure how deadly something is [34]. It is a safe and cheap way to find out if a chemical or plant product is harmful to cells [52]. It is also the primary tool for detecting antitumor properties [34]. Their LC_50_ values can evaluate the toxicity of plant extracts. If LC_50_ values are lower than 1000 *μ*g/mL, it is cytotoxic [34]. In contrast, LC_50_ values of stem and seed BO showed 39.199 *µ*g/mL and 10.659 *µ*g/mL chronologically and standard vincristine sulfate showed 0.84 *µ*g/mL (Table 5). Despite having lower activity than vincristine sulfate, those two components of BO extract can be considered potent cytotoxic drug holders.

Some studies confirmed that cytotoxic activity is responsible for secondary metabolites such as phenolics, flavonoids, and terpenoids [53–59]. The presence of terpenoids in the stem extract may explain why the stem of BO is more potent than the seed. The stem and seed of BO also contain flavonoids and phenols, which may also be responsible for exerting this cytotoxic effect.

### 3.6. Thrombolytic Activity In Vitro

Thrombolysis, also called thrombolytic therapy, is used to break up large blood clots, get blood flowing again, and protect organs and tissues from damage. Thrombolytic drugs break up blood clots by turning on plasminogen, which causes plasmin to split into two parts. Plasmin is a proteolytic enzyme that may break cross-links between fibrin molecules, which are responsible for the structural stability of blood clots.

The results of the percentage of clot lysis of the ethanolic extracts of different parts of BO (stem and seed), the positive control (streptokinase), and the negative control (water) are shown in Figure 1. For positive control (100 *μ*L of streptokinase 30, 000 IU), the clot lysis was 76.15 ± 1.94% and 92.59 ± 2.35% after 1.5 h and 24 h incubation. In contrast, in the case of negative control (water), the clot lysis was found at 3.59 ± 0.46% and 9.14 ± 1.05% after 1.5 h and 24 h of incubation, respectively.

**Figure 4:**
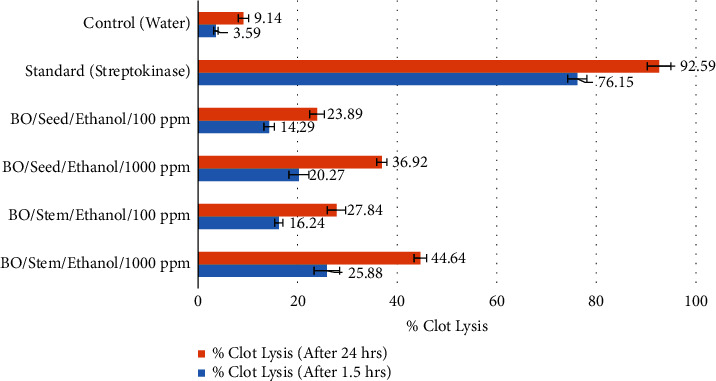
Thrombolytic properties of ethanolic extracts of stem and seed of BO compared with standard (streptokinase).

It is seen from Figure 4 that 1000 ppm and 100 ppm solution of the seed of BO showed 20.27 ± 2.01 and 14.29 ± 1.03 after 1.5 hours and 36.92 ± 1.02 and 23.89 ± 1.49 after 24 hours of incubation period, respectively. Again, the concentration of the stem of BO was 1000ppm and 100ppm, percent clot lysis was 25.88 ± 2.01 and 16.24 ± 1.03 after 1.5 h and 44.64 ± 1.02 and 27.84 ± 1.49 after 24 h incubation time respectively.

Some studies indicate that thrombolytic activity is probably due to the diverse composition of plant extracts' phytoconstituents, including alkaloids, flavonoids, tannins, and terpenoids [60, 61]. The stem of BO is more active compared with the seed of BO. It may be due to the presence of terpenoids in the stem extract.

### 3.7. In Vitro Antiarthritic Activity

Antiarthritic medicine treats or prevents arthritic symptoms such as joint pain and stiffness. Depending on the type of antiarthritis drug, it may help in pain, reduce inflammation, or weaken the immune system. Denaturation of proteins may contribute to the production of auto antigens in some arthritic conditions. Changes to electrostatic, hydrogen, hydrophobic, and disulfide bonds are likely part of the denaturation pathway [62].

**Figure 5:**
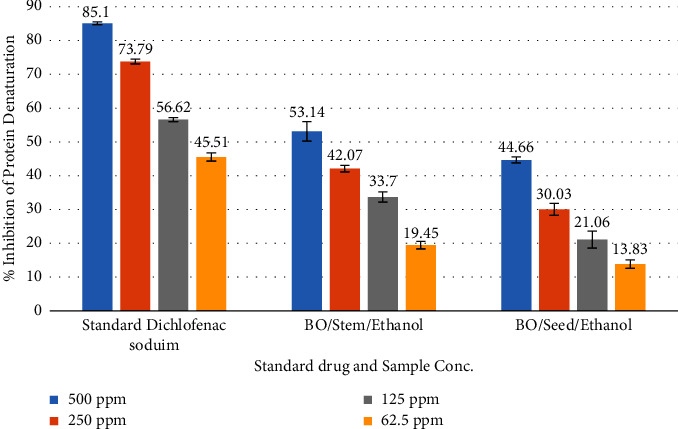
Antiarthritic activity of ethanolic extracts of BO stem and seed compared to standard (diclofenac sodium).

In vitro antiarthritic activities of ethanolic extract of seed and stem of BO were observed.  Figure 5 represents the data on the antiarthritic activities of stem and seed of BO plant extracts. The percent inhibition of protein denaturation of 500 ppm plant extract of the stem and the seed of BO showed 53.14 ± 2.87% and 44.66 ± 0.89%, respectively, and standard showed the maximum of 85.10 ± 0.45%. A 250 ppm plant extract of the stem and seed of BO inhibited protein denaturation by 42.07 ± 0.94% and 30.03 ± 1.76%, respectively, while the standard inhibited protein denaturation by 73.79 ± 0.73%.

The percent inhibition of protein denaturation of 125 ppm plant extract of stem and seed of BO showed 33.70 ± 1.53% and 21.06 ± 2.52%, respectively, chronologically and standard showed 56.62 ± 0.56. The inhibition of protein denaturation of 62.5 ppm plant extract of stem and seed of BO showed 19.45 ± 1.08% and 13.83 ± 1.27%, respectively, and standard showed a minimum of 45.51 ± 1.21%.

Some phytochemical constituents have the same properties, such as flavonoids, which have antirheumatism, antihypertensive, antimicrobial, diuretic, and antioxidant properties. Some phytochemicals have the same effects. For example, flavonoids can treat rheumatism, high blood pressure, infections, make you pee, and fight free radicals [63, 64]. Both the stem and the seed of BO contain flavonoids, but the stem may contain a comparatively higher amount of flavonoids than the seed according to its activity.

The results of our study show that extracts of BO could be used as antimicrobials, in chemotherapy, to break up blood clots, and to stop the body from making autoantibodies. Our research shows that extracts from different parts of the BO have low to moderate antimicrobial, cytotoxic, thrombolytic, and antiarthritic activity. Still, these could be an important source of antibacterial, antifungal, anticancer, antitrauma, thrombolytic, and antiarthritic elements.

## 4. Conclusion

In this study, 14 microorganisms were used, and orchid samples were compared to ciprofloxacin as a reference. It was discovered that the BO/seed extract had significant antibacterial activity, compared to BO/stem extract. The LC_50_ values were evaluated using the brine shrimp lethality assay. The BO/stem extract demonstrated a more destructive effect than the BO/seed extract, measuring LC_50_ value for 10.659 *μ*g/mL. To evaluate the extracts' thrombolytic activity, two concentrations (1000 and 100 ppm) and two incubation periods (24 and 1.5 hours) were used. In terms of its thrombolytic activity, the BO/stem extract had shown more potential. Furthermore, the highest values for protein denaturation were found at 500, 250, 125, and 62.5 ppm (53.14 ± 2.87%, 42.07 ± 0.94%, 33.70 ± 1.53, and 19.45 ± 1.08%), indicating the antiarthritic efficacy of the BO/stem extract to other parts. Our study will be helpful to find novel phytochemicals in light of the emergence of drug-resistant microorganisms, cancer, stroke due to blood clots, autoimmune disease, etc. Positive results obtained from different investigations mentioned above run on different organs of orchid were provided us a primary indication of its effectiveness as antimicrobial agents, antitumor, antiarthritic, and thrombolytic compounds. Further research on this plant is needed. This study could play a vital role in finding a lot of sources to invent drugs for the treatment of diseases related to antimicrobial, cytotoxic, thrombolytic, and arthritic activity as candidates for the future in vivo investigation.

## Figures and Tables

**Table 1:** Different phytochemicals found in ethanolic extracts of different parts of BO.

Sample name	Chemical group test									
	Saponin	Steroid	Glycoside	Terpenoid	Carbohydrate	Phenol	Flavonoid	Tannin	Alkaloid	Protein
BO/seed/EtOH	−	+	+	−	+	+	+	−	+	+
BO/stem/EtOH	−	+	+	+	+	+	+	−	+	+

BO indicates *Bari orchid-1* and (+) indicates presence, (−) indicates absence.

**Table 2:** UV-visible peaks of different plant extracts.

Plant name, part of the plant, and extracted medium	Wavelength (nm)
BO/seed/ethanol	242.1
BO/stem/ethanol	235.4, 327.5, 401.5, 665.4

**Table 3:** FTIR peaks of two different parts of BO extracts.

Plant name, part of the plant, and extracted medium	Wavenumbers (cm^−1^)
BO/stem/ethanol	881, 1023, 1449, 2832, 2943, 3324
BO/seed/ethanol	881, 1023, 1449, 1654, 2832, 2944, 3325

**Table 4:** Antimicrobial activity of ethanolic extracts of different parts of BO.

Microbiological test			
Name of microorganism	Name of standard/sample (ethanolic extract)		
	Standard Ciprofloxacin (mm)	Stem of BO (mm)	Seed of BO (mm)
*Gram-positive bacteria*			
*Bacillus cereus*	45	10	13
*Bacillus megaterium*	45	12	14
*Bacillius subtilis*	45	10	13
*Staphylococcus aureus*	46	12	14
*Sarcina lutea*	45	10	14

*Gram-negative bacteria*			
*Salmonella paratyphi*	43	12	13
*Salmonella typhi*	45	10	10
*Vibrio parahaemolyticus*	45	12	14
*Vibrio mimicus*	46	10	12
*Escherichia coli*	44	10	12
*Shigella dysenteriae*	46	10	14
*Pseudomonas aeruginosa*	45	10	14
*Shigella boydii*	45	10	14

*Fungi*			
*Saccharomyces cerevisiae*	45	10	14
*Candida albicans*	45	10	14
*Aspergillus niger*	45	10	14

**Table 5:** Brine shrimp lethality bioassay of ethanolic extracts of different parts of BO.

Sample name	LC_50_ (*μ*g/mL)
BO/Seed/EtOH	39.199
BO/Stem/EtOH	10.659
Vincristine sulfate (standard)	0.84

## Data Availability

The data used to support the findings of this study are included within the article.

## References

[1] Tandon V., Gupta R. K. (2004). Histomorphological changes induced by Vitex negundo in albino rats. *Indian Journal of Pharmacology*.

[2] Vispute S., Khopade A. (2011). Glycyrrhiza glabra Linn.- ‘Klitaka’: a review. *International Journal of Pharma and Bio Sciences*.

[3] Pankti K., Payal G., Manodeep C., Jagadish K. (2012). A phytopharmacological review of Alstonia scholaris: a panaromic herbal medicine. *International Journal of Research in Ayurveda and Pharmacy*.

[4] Jalal J. S., Kumar P., Pangtey Y. P. S. (2008). Ethnomedicinal orchids of uttarakhand, western himalaya. *Ethnobotanical Leaflets*.

[5] Mia M. A. B. (2003). Bari orchid 1. *Digital Herbarium of Crop Plants*.

[6] Sample I. (2018). Calls to rein in antibiotic use after study shows 65% increase worldwide. https://www.theguardian.com/science/2018/mar/26/calls-to-rein-in-antibiotic-use-after-study-shows-65-increase-worldwide.

[7] World Health Organization (2014). *Antimicrobial Resistance: Global Report on Surveillance*.

[8] O’Neill J. (2016). *Tackling Drug-Resistant Infections Globally: Final Report and Recommendation in the Review on Antimicrobial Resistance*.

[9] World Health Organization (2020). Lack of new antibiotics threatens global efforts to contain drug-resistant infections. https://www.who.int/news-room/detail/17-01-2020-lack-of-new-antibiotics-threatens-global-efforts-to-contain-drug-resistant-infections.

[10] Iwu M. W., Duncan A. R., Okunji C. O. (1999). *New Antimicrobials of Plant Origin. Perspectives on New Crops and New Uses*.

[11] World Health Organization (2002). *WHO Traditional Medicine Strategy 2002-2005*.

[12] Coates A., Hu Y., Bax R., Page C. (2002). The future challenges facing the development of new antimicrobial drugs. *Nature Reviews Drug Discovery*.

[13] Marasini B. P., Baral P., Aryal P. (2015). Evaluation of antibacterial activity of some traditionally used medicinal plants against human pathogenic bacteria. *BioMed Research International*.

[14] Evans N. S., Ratchford E. V. (2014). Vascular disease patient information page: venous thromboembolism (deep vein thrombosis and pulmonary embolism). *Vascular Medicine*.

[15] Benjamin E. J., Virani S. S., Callaway C. W. (2018). Heart disease and stroke statistics—2018 update: a report from the American Heart Association. *Circulation*.

[16] Ali M., Salim Hossain M., Islam M. (2014). Aspect of thrombolytic therapy: a review. *The Scientific World Journal*.

[17] Verstraete M. (2000). Third-generation thrombolytic drugs. *The American Journal of Medicine*.

[18] Chen C., Yang F. Q., Zhang Q., Wang F. Q., Hu Y. J., Xia Z. N. (2015). Natural products for antithrombosis. *Evidence-based Complementary and Alternative Medicine*.

[19] Murugananthan G., Mohan S. (2013). Anti-arthritic and anti-inflammatory constituents from medicinal plants. *Journal of Applied Pharmaceutical Science*.

[20] Mazumder P. M., Mondal A., Sasmal D., Arulmozhi S., Rathinavelusamy P. (2012). Evaluation of antiarthritic and immunomodulatory activity of Barleria lupulina. *Asian Pacific Journal of Tropical Biomedicine*.

[21] Rader D. J., Kathiresan S., Jameson J., Fauci A. S., Kasper D. L., Hauser S. L., Longo D. L. (2018). Disorders of lipoprotein metabolism.

[22] Rajkapoor B., Ravichandran V., Gobinath M. (2007). Effect of Bauhinia variegata on complete Freund`s adjuvant induced arthritis in rats. *Journal of Pharmacology and Toxicology*.

[23] Chitme H. R., Patel N. P. (2009). Antiarthritis activity of Aristolochia bracteata extract in experimental animals. *The Open Natural Products Journal*.

[24] R Hamidi M., Jovanova B., Kadifkova Panovska T. (2014). Toxicological evaluation of the plant products using Brine Shrimp (*Artemia salina* L.) model. *Macedonian Pharmaceutical Bulletin*.

[25] Saadabi A. M. (2006). Antifungal activity of some Saudi plants used in traditional medicine. *Asian Journal of Plant Sciences*.

[26] Olowa L. F., Nuñeza O. M. (2013). Brine shrimp lethality assay of the ethanolic extracts of three selected species of medicinal plants from Iligan City, Philippines. Mortality, 1(T2), T3. *International Research Journal of Biological Sciences*.

[27] Evans W. C. (2009). *Trease and Evans’ Pharmacognosy*.

[28] Harborne J. B. (1998). *Textbook of Phytochemical Methods. A Guide to Modern Techniques of Plant Analysis*.

[29] Rahaman M. S., Rahaman M. S., Bari M. A. (2019). An approach to evaluate anti-arthritic and thrombolytic activity of different parts of solanum torvum sw. (solanaceae) and smilax zeylanica L. (Liliaceae). *Journal of Drug Delivery and Therapeutics*.

[30] Daoud A., Malika D., Bakari S. (2019). Assessment of polyphenol composition, antioxidant and antimicrobial properties of various extracts of Date Palm Pollen (DPP) from two Tunisian cultivars. *Arabian Journal of Chemistry*.

[31] Bauer A. W., Kirby W. M., Sherris J. C., Turck M. (1966). Antibiotic susceptibility testing by a standardized single disk method. *American Journal of Clinical Pathology*.

[32] Raju G. S., Moghal M. R., Dewan S. M. R., Amin M. N., Billah M. (2013). Characterization of phytoconstituents and evaluation of total phenolic content, anthelmintic, and antimicrobial activities of Solanum violaceum Ortega. *Avicenna Journal of Phytomedicine*.

[33] Wilkinson J. M., Ahmad I., Aqil F., Owais M. (2007). Methods for testing the antimicrobial activity of extracts. *Modern Phytomedicine: Turning Medicinal Plants into Drugs*.

[34] Meyer B. N., Ferrigni N. R., Putnam J. E., Jacobsen L. B., Nichols D. E. J., McLaughlin J. L. (1982). Brine shrimp: a convenient general bioassay for active plant constituents. *Planta Medica*.

[35] McLaughlin J. L., Rogers L. L., Anderson J. E. (1998). The use of biological assays to evaluate botanicals. *Drug Information Journal*.

[36] Partricia M., Fiona S., Salem A. Q. (2005). Antioxidant, antibacterial activities and general toxicity of Alnus glutinosa, fraxinus excelsior and papaver rhoeas. *Iranian Journal of Pharmaceutical Research*.

[37] Persoone G., Sorgeloos P., Roels O., Jaspers E. (1979). The brine shrimp Artemia. *Proceedings of the International Symposium on the Brine Shrimp Artemia salina*.

[38] Apu A. S., Muhit M. A., Tareq S. M., Pathan A. H., Jamaluddin A. T. M., Ahmed M. (2010). Antimicrobial activity and brine shrimp lethality bioassay of the leaves extract of Dillenia indica linn. *Journal of Young Pharmacists*.

[39] Prasad S., Kashyap R. S., Deopujari J. Y., Purohit H. J., Taori G. M., Daginawala H. F. (2007). Effect of Fagonia arabica (Dhamasa) on in vitro thrombolysis. *BMC Complementary and Alternative Medicine*.

[40] Prasad S., Kashyap R. S., Deopujari J. Y., Purohit H. J., Taori G. M., Daginawala H. F. (2006). Development of an in vitro model to study clotlysis activity of thrombolytic drugs. *Thrombosis Journal*.

[41] Pavithra T. K., Smitha K. P., Kulashekar K. S., Ashok Kumar B. S. (2015). Evaluation of in vitro anti-arthritic activity of vitex negundo against the denaturation of protein. *International Journal of Current Microbiology and Applied Sciences*.

[42] Jain P. K., Soni A., Jain P., Bhawsar J. (2016). Phytochemical analysis of Mentha spicata plant extract using UV-VIS, FTIR and GC/MS technique. *Journal of Chemical and Pharmaceutical Research*.

[43] Tošović J., Milošević Ž., Marković S. (2015). Simulation of the UV/Vis spectra of flavonoids. *2015 IEEE 15th International Conference on Bioinformatics and Bioengineering (BIBE)*.

[44] Moses A. G. M., Robert M. N. (2013). FourierTransform infra-red spectrophotometer analysis of warburgia ugandensis medicinal herb used for the treatment of diabetes, malaria and pneumonia in kisii region, southwest Kenya. *Global Journal of Pharmacology*.

[45] Doughari J. H. (2007). Antimicrobial activity of *Tamarindus indica* linn. *Tropical Journal of Pharmaceutical Research*.

[46] Kiani Y. S., Jabeen I. (2019). Lipophilic metabolic efficiency (LipMetE) and drug efficiency indices to explore the metabolic properties of the substrates of selected cytochrome P450 isoforms. *ACS Omega*.

[47] Yuan G., Guan Y., Yi H., Lai S., Sun Y., Cao S. (2021). Antibacterial activity and mechanism of plant flavonoids to gram-positive bacteria predicted from their lipophilicities. *Scientific Reports*.

[48] Nugraha A. S., Triatmoko B., Wangchuk P., Keller P. A. (2020). Vascular epiphytic medicinal plants as sources of therapeutic agents: their ethnopharmacological uses, chemical composition, and biological activities. *Biomolecules*.

[49] Raghavanpillai Sabu K., Sugathan S., Idhayadhulla A. (2022). Antibacterial, antifungal, and cytotoxic activity of *Excoecaria agallocha* leaf extract. *Journal of Experimental Pharmacology*.

[50] Abate L., Bachheti A., Bachheti R. K., Husen A. (2021). Antibacterial properties of medicinal plants: recent trends, progress, and challenges. *Traditional Herbal Therapy for the Human Immune System*.

[51] Sieberi B. M., Omwenga G. I., Wambua R. K., Samoei J. C., Ngugi M. P. (2020). Screening of the dichloromethane: methanolic extract of *Centella asiatica* for antibacterial activities against *Salmonella typhi*, *Escherichia coli*, Shigella sonnei, Bacillus subtilis, and *Staphylococcus aureus*. *The Scientific World Journal*.

[52] de Almeida P. A., da Silva T., Echevarria A. (2002). Mesoionic 5-ALKYL-1,3-DITHIOLIUM-4-THIOLATES: synthesis and brine shrimp toxicity. *Heterocyclic Communications*.

[53] Anderson J. E., Goetz C. M., McLaughlin J. L., Suffness M. (1991). A blind comparison of simple bench‐top bioassays and human tumour cell cytotoxicities as antitumor prescreens. *Phytochemical Analysis*.

[54] Coe F. G., Parikh D. M., Johnson C. A. (2010). Alkaloid presence and brine shrimp (*Artemia salina*) bioassay of medicinal species of eastern Nicaragua. *Pharmaceutical Biology*.

[55] Vila R., Iglesias J., Cañigueral S., Santana A. I., Solís P. N., Gupta M. P. (2004). Constituents and biological activity of the essential oil of Eugenia acapulcensis Steud. *Journal of Essential Oil Research*.

[56] Lee K. H. (1992). Plant phenolic compounds as cytotoxic antitumor agents. *ACS Symposium Series*.

[57] Ren W., Qiao Z., Wang H., Zhu L., Zhang L. (2003). Flavonoids: promising anticancer agents. *Medicinal Research Reviews*.

[58] Tungmunnithum D., Thongboonyou A., Pholboon A., Yangsabai A. (2018). Flavonoids and other phenolic compounds from medicinal plants for pharmaceutical and medical aspects: an overview. *Medicine (Baltimore)*.

[59] Aryal S., Baniya M. K., Danekhu K., Kunwar P., Gurung R., Koirala N. (2019). Total phenolic content, flavonoid content and antioxidant potential of wild vegetables from western Nepal. *Plants*.

[60] Dwivedi S. (2007). Terminalia arjuna Wight & Arn.--a useful drug for cardiovascular disorders. *Journal of Ethnopharmacology*.

[61] Sherwani S. K., Khan M. M., Zubair A., Shah M. A., Kazmi S. U. (2013). Evaluation of in vitro thrombolytic activity of Bougainvillea spectabilis leaf extract. *International Journal of Pharmaceutical Sciences Review and Research*.

[62] Arya D., Meena M., Neha G., Vidya P. (2014). In vitro anti-inflammatory and anti-arthritic activity in methanolic extract of Cocculus hirsutus (L.) Diels. In vovo and in vitro. *Int J Pharm Sci*.

[63] Burkill H. M. (1986). *The Useful Plants of West Tropical Africa*.

[64] Trease G. E., Evans W. E. (2002). *Pharmacognos*.

